# Nabilone for non-motor symptoms of Parkinson’s disease: a randomized placebo-controlled, double-blind, parallel-group, enriched enrolment randomized withdrawal study (The NMS-Nab Study)

**DOI:** 10.1007/s00702-019-02021-z

**Published:** 2019-05-25

**Authors:** Marina Peball, Mario Werkmann, Philipp Ellmerer, Raphaela Stolz, Dora Valent, Hans-Günther Knaus, Hanno Ulmer, Atbin Djamshidian, Werner Poewe, Klaus Seppi

**Affiliations:** 1 Department of Neurology, Innsbruck Medical University, Anichstrasse 35, 6020 Innsbruck, Austria; 2 Department for Medical Genetics, Molecular and Clinical Pharmacology, Innsbruck Medical University, Peter-Mayr Straße 1, 6020 Innsbruck, Austria; 3 Department of Medical Statistics, Informatics and Health Economics, Innsbruck Medical University, Schöpfstraße 41/1, 6020 Innsbruck, Austria

**Keywords:** Parkinson’s disease, Non-motor symptoms, Cannabinoids, Nabilone

## Abstract

Although open-label observations report a positive effect of cannabinoids on non-motor symptoms (NMS) in Parkinson’s disease (PD) patients, these effects remain to be investigated in a controlled trial for a broader use in NMS in PD patients. Therefore, we decided to design a proof-of-concept study to assess the synthetic cannabinoid nabilone for the treatment of NMS. We hypothesize that nabilone will improve NMS in patients with PD and have a favorable safety profile. The NMS-Nab Study is as a mono-centric phase II, randomized, placebo-controlled, double-blind, parallel-group, enriched enrollment withdrawal study. The primary efficacy criterion will be the change in Movement Disorders Society-Unified Parkinson’s Disease-Rating Scale Part I score between baseline (i.e. randomization) and week 4. A total of 38 patients will have 80% power to detect a probability of 0.231 that an observation in the treatment group is less than an observation in the placebo group using a Wilcoxon rank-sum test with a 0.050 two-sided significance level assuming a true difference of 2.5 points between nabilone and placebo in the primary outcome measure and a standard deviation of the change of 2.4 points. The reduction of harm through an ineffective treatment, the possibility of individualized dosing, the reduction of sample size, and the possible evaluation of the influence of the placebo effect on efficacy outcomes justify this design for a single-centered placebo-controlled investigator-initiated trial of nabilone. This study should be the basis for further evaluations of long-term efficacy and safety of the use of cannabinoids in PD patients.

## Background

Although Parkinson’s disease (PD) is generally considered a paradigmatic movement disorder, it has long been recognized that the neuropathology underlying PD involves many brain areas that are not directly involved in motor control (Braak et al. 2003). It is, therefore, not surprising that a majority, if not all, PD patients suffer from a variety of different non-motor symptoms (NMS) adding to the overall burden of parkinsonian morbidity (Hely et al. 2005, 2008; Poewe 2006; Poewe et al. 2017). NMS in PD involve a multitude of functions including disorders of sleep–wake cycle regulation, cognitive dysfunction, disorders of mood and affect, autonomic dysfunction as well as sensory symptoms and pain. They become increasingly prevalent and obvious over the course of the illness and are a major determinant of quality of life (QoL), progression of overall disability, and of nursing home placement in PD (Pfeiffer 2016). Common NMS in late stage PD include hallucinations, dementia, and autonomic dysfunction including urinary incontinence, constipation, and symptomatic orthostatic hypotension (Hely et al. 2008).

Despite increasing awareness in recent years, NMS in PD are still frequently missed or undeclared during routine consultations and well-performed large-scale randomized controlled trials (RCTs) for the treatment of the different NMS in PD are lacking (Schrag et al. 2015; Seppi et al. 2011, 2019). Therefore, unlike most motor features of PD, treatment options for NMS are limited and outcomes are often unsatisfactory (Kalia and Lang 2015).

## Scientific rationale for cannabinoids in Parkinson’s disease

### The endocannabinoid system (ECS) and PD

Data from animal studies reveal a high density of cannabinoid (CB) receptors in the basal ganglia where CB1 receptors are co-localised with striatal dopaminergic receptors and both receptor types are involved in common signaling pathways (Brotchie 2003; Mursaleen and Stamford 2016; Pacher et al. 2006). The majority of CB1 receptors is localized on striatal glutamatergic and gamma-aminobutyric acid (GABA)-ergic interneurons and projection neurons. CB1 receptor stimulation leads to a decrease in glutamatergic drive in the striatum and is believed to contribute to motor learning and behavior via modulation of glutamatergic synaptic plasticity (Brotchie 2003; Mursaleen and Stamford 2016; Pacher et al. 2006). Postsynaptic striatal GABA-ergic neurons mostly release endocannabinoids to cause retrograde depression of neurotransmitter release in the glutamatergic neurons (Brotchie 2003; Mursaleen and Stamford 2016; Pacher et al. 2006).

Studies in PD indicate that the ECS is overactive with increase in its neurotransmitter (e.g. anandamide), CB1 receptor levels, and CB1-receptor-G-protein coupling within the striatum following loss of dopamine (Brotchie 2003; Pacher et al. 2006). Stimulation of CB1 receptors in the internal and external globus pallidus (GPi, GPe) leads to decreased GABA uptake and a consequential inhibition of the GPi and GPe with motor depression to follow (Brotchie 2003; Mursaleen and Stamford 2016; Pacher et al. 2006).

### The endocannabinoid system (ECS) and NMS

Because of its (neuro)modulatory effects, the ECS has been a center of attention in the last 25 years and has become a potential target of drug therapy for a variety of illnesses. The ECS plays an important role in the regulation of motor control as well as of many non-motor functions including mood, attention and concentration, eating behavior, sleep, and nociception (Castillo et al. 2012; Kluger et al. 2015). The specific underlying mechanisms by which the ECS influences emotion and pain processing, as well as sleep remain unclear. So far, evidence from preclinical and clinical studies suggests an alteration of endocannabinoid signaling in response to pain and psychiatric symptoms. In animal studies, a high density of CB1 receptors is found in presynaptic nerve terminals of GABA-ergic synapses as well as neurons with μ-opioid receptors in the cortex and limbic areas of the brain, both of which are responsible for processing of emotion and nociception (Chiou et al. 2013; Fitzgibbon et al. 2015). In line with this, nociceptive pathways in the dorsal spinal cord are surrounded by structures of the ECS (e.g. CB1 receptors) that contribute to analgesia (Chiou et al. 2013; Fitzgibbon et al. 2015; Huang et al. 2016). Furthermore, serotoninergic, noradrenergic, and dopaminergic neurons express a high amount of CB1 receptors that influence monoaminergic neurotransmission, e.g. in the procession of pain. The modulation of serotoninergic and noradrenergic neurons by cannabinoids in the central nervous system and spinal cord can, therefore, contribute to the positive effects of cannabinoids on pain and mood-related symptoms (Fitzgibbon et al. 2015; Huang et al. 2016). Clinical studies have shown decreased serum levels of endocannabinoids in patients with depression and chronic pain (Fitzgibbon et al. 2015). Studies in patients with human immunodeficiency virus (HIV), cancer, fibromyalgia, posttraumatic stress disorder, diabetic peripheral neuropathic pain and central neuropathic pain in multiple sclerosis revealed an improvement of pain, depression, and anxiety with the use of cannabinoids (Fitzgibbon et al. 2015; Maida et al. 2008; Rog et al. 2007; Skrabek et al. 2008; Toth et al. 2012; Weber et al. 2009; Williamson and Evans 2000; Woolridge et al. 2005). Moreover, patients with chronic pain and psychiatric diseases (e.g. major depression and bipolar disorder) showed genetic polymorphisms of CB1 and CB2 receptors, which were in turn associated with resistance to treatment of depression (Fitzgibbon et al. 2015; Huang et al. 2016). Thus, pain in PD patients may be associated with single-nucleotide polymorphisms in a cannabinoid-metabolizing enzyme (Greenbaum et al. 2012).

The few studies assessing the ECS and sleep revealed that exogenous cannabinoids promote sleep, increase rapid eye movement (REM) sleep and the stability of non-REM (NREM) sleep, although the precise underlying pathophysiological mechanisms remain unclear (Murillo-Rodriguez 2008; Murillo-Rodriguez et al. 2003; Pava et al. 2016).

Taken together, these preclinical and clinical data demonstrate that cannabinoids might modulate nociception, influence mood and emotional processing, as well as sleep in spinal and supra-spinal regions.

### Clinical trials of cannabinoids in PD

Evidence of the effect of cannabinoids on symptoms in PD from clinical trials is scarce. Most of these trials are either small-sized with less than 20 patients included (Carroll et al. 2004; Kluger et al. 2015; Sieradzan et al. 2001) or uncontrolled (Balash et al. 2017; Chagas et al. 2014a; Kluger et al. 2015; Lotan et al. 2014; Venderova et al. 2004; Zuardi et al. 2009). Improvement of motor and NMS in PD after intake of cannabinoids has been described in several uncontrolled observational studies with ameliorated rest tremor, bradykinesia, rigidity, and levodopa-induced dyskinesia (LID) as well as improved pain, depression, psychosis, hallucinations, orientation, symptoms of RBD, and sleep quality (Balash et al. 2017; Chagas et al. 2014a; Lotan et al. 2014; Venderova et al. 2004; Zuardi et al. 2009). Currently, no data on the use of cannabinoids to treat NMS in PD patients are available from randomized controlled trials (RCTs). RCTs assessing QoL in PD patients using cannabinoids either reported of an improvement (Chagas et al. 2014b) or no benefit (Carroll et al. 2004). Regarding motor symptoms of PD, one RCT found an amelioration of LID in response to a CB1 receptor agonist, while others found no difference in LID or the total UPDRS scores between patients and the placebo group (Sieradzan et al. 2001).

Cannabinoids were well tolerated in all of these trials. No serious adverse events were recorded. The most common side effects from the RCTs have been the feeling of dizziness in up to 10% of patients, mild hypotension in up to 90% of patients, deterioration or new-onset change of perception (hallucinations, the feeling to be detached, confusion), and somnolence in up to 47% of patients (Carroll et al. 2004; Chagas et al. 2014b; Mesnage et al. 2004; Sieradzan et al. 2001). In one RCT, two patients who received nabilone withdrew from the study due to vertigo and postural hypotension (Sieradzan et al. 2001) but this was not consistent with other RCTs (Carroll et al. 2004; Chagas et al. 2014b) which included a larger sample size and documented no withdrawals due to side effects of cannabinoids.

### Rationale for the use of nabilone in PD patients

The study drug nabilone is an analog of tetrahydrocannabinol (THC), the psychoactive component of cannabis, but it is not directly derived from the cannabis plant. Nabilone acts as a partial agonist on both CB1 and CB2 receptors in humans and, therefore, mimics the effect of THC but with more predictable side effects and less euphoria. Nabilone is commercially available and its use is usually safe and well tolerated. In Austria, nabilone is licensed for the use as an antiemetic for chemotherapy-induced nausea and vomiting not responding to conventional antiemetic treatment. The United States Food and Drug Administration (FDA) approved nabilone for treating anorexia and weight loss in patients with acquired immune deficiency syndrome (AIDS). Nabilone is used as an adjunct therapy for chronic pain management (Lynch and Campbell 2011; Turcotte et al. 2015), although it is only officially approved for this use in Mexico.

To our current understanding, endogenous and exogenous cannabinoids such as nabilone may improve sleep, and alleviate pain and mood disorders via modulation of monoaminergic, GABA-ergic, glutamatergic neurons and opioid signaling in nociception and mood processing. Although data from observational studies report a positive effect of cannabinoids on NMS in PD patients, these effects remain to be investigated in a controlled trial for a broader use in NMS in PD patients.

### Addictive potential of nabilone

The potential for abuse of nabilone was deemed low and unlikely. There is very little evidence of recreational use of nabilone which is believed to be mostly due to its effect profile (e.g. less euphoria), high costs, slower onset of action, and more difficult titration compared to smoking cannabis (Ware and St Arnaud-Trempe 2010).

On the contrary, nabilone has been used in a small number of patients with cannabis dependence and has shown to reduce marijuana use per se as well as withdrawal symptoms (Haney et al. 2013).

However, it is recommended to evaluate patients using cannabinoids regularly for effects of tolerance and dependency (Ware and St Arnaud-Trempe 2010).

## Hypothesis

Due to the overall impact of NMS in PD and based on preclinical and clinical data on the influence of the endocannabinoid system on nociception, emotions, and sleep, we decided to design a proof-of-concept study to assess the synthetic cannabinoid nabilone for the treatment of NMS in PD. We hypothesize given the data and possible modes of action of the endocannabinoid system that nabilone will improve NMS in patients with PD and will have a favorable safety profile.

## Methods/design

### Subjects

In this trial, eligible male and female PD patients with stable motor disease, i.e. without disturbing motor fluctuations or dyskinesia according to the Movement Disorders Society-Unified Parkinson’s Disease-Rating Scale (MDS-UPDRS) Part IV over the age of 30 years suffering from NMS (measured on the basis of MDS-UPDRS I, including at least anxiety or pain) will be included. Eligibility will be assessed by inclusion and exclusion criteria (Table 1). To participate in the study, patients must have a score of at least ≥ 4 points on MDS-UPDRS Part 1 with ≥ 2 points in the item for anxiety or pain. Patients with disturbing impulse control disorders as defined per cut-off values of the Questionnaire for Impulsive-Compulsive Disorders in Parkinson’s Disease-Rating Scale (QUIP-RS) will be excluded (Probst et al. 2014). Furthermore, PD patients with symptomatic orthostatic hypotension, sinus tachycardia, and chronic major psychiatric disorders will be excluded as these are possibly dangerous adverse reactions that may occur during the intake of nabilone. Patients with moderately or severely impaired liver function and/or chronic alcohol or drug abuse will be excluded from the trial because the primary route of elimination of nabilone is biliary. As this study is a pilot study, we decided to reduce bias as much as possible by applying these inclusion and exclusion criteria. Moreover, some of the exclusion criteria are applied for safety reasons such as exclusion of patients with severe depression, severe hallucinations, severe symptomatic orthostatic hypotension, severe cognitive impairment, or severe impulse control disorders.

**Table 1:** Inclusion and exclusion criteria

Inclusion criteria	Exclusion criteria
1. Age ≥ 30 years 2. Diagnosis of PD: PD should be either de novo or on stable medication without disturbing motor fluctuations or dyskinesia 3. NMS with a score of ≥ 4 on MDS-UPDRS Part 1. One of the following domains has to be affected with a score ≥ 2: 1.4 (anxious mood) or 1.9 (pain) 4. On a stable regimen of anti-parkinson medications for at least 30 days prior to screening and willing to continue the same doses and regimens during study participation 5. Any other current and allowed prescription/non-prescription medications and/or nutritional supplements taken regularly must have been at a stable dose and regimen for at least 30 days prior to screening, and subject must be willing to continue the same doses and regimens during study participation 6. Patient is informed and had enough time and opportunity to think about his/her participation in the study and has signed a current IRB-approved informed consent form 7. Contraception (a) Women of child-bearing potential must use or attest an acceptable method of contraception starting 4 weeks prior to study drug administration and for a minimum of 1 month after study completion (b) Men with a potentially fertile partner must be willing to use an acceptable method of contraception for the duration of the study and for 3 months after study drug discontinuation or have had a vasectomy	1. Patient previously participated in any study with nabilone 2. Current use of cannabinoids or use of cannabinoids within 30 days prior to screening 3. Patient is currently participating in or has participated in another study of investigational products within 30 days prior to screening 4. Patient has any form of secondary or atypical parkinsonism (e.g. drug induced, post stroke) 5. Patient presents with motor complications which are, based on the investigator’s judgment, not adequately controlled (i.e. a score ≥ 2 on one of the items of the MDS-UPDRS Part IV at screening) 6. Hoehn and Yahr stage > 3 7. Evidence of disturbing (i.e. requiring treatment) impulse control disorder in the participant. Can be resolved through a structural interview during screening period 8. History of neurosurgical intervention for PD 9. The presence of symptomatic orthostatic hypotension at screening (MDS-UPDRS 1.12 > 2) 10. Use of prohibited medication as listed in the protocol 11. Patients with laboratory values that are out of range at screening (or within 4 weeks prior to screening) and have not been reviewed and documented as not clinically significant by the investigator. Lab tests can be repeated for confirmation 12. Patients with known or newly diagnosed sinus tachycardia in ECG evaluation at screening or within 4 weeks prior to screening 13. The presence of an acute or chronic major psychiatric disorder (e.g. major depressive disorder, psychosis) or symptom (e.g. hallucinations, agitation, paranoia) (MDS-UPDRS 1.2 and/or 1.3 > 2) 14. Patients who had a recent suicidal attempt (active, interrupted, aborted) within the past 5 years or report suicidal ideation within the past 6 months 15. The presence of dementia (MDS-UPDRS 1.1 > 2, MMSE of < 24 at the screening visit) 16. Clinically significant or unstable medical or surgical condition at screening or baseline visit that may preclude safety and the completion of the study participation (based on the investigator’s judgment) 17. Patients with moderate or severe hepatic or renal impairment 18. Patient has a history of chronic alcohol or drug abuse within the last 2 years 19. Women of child-bearing potential who do not practice an acceptable method of birth control 20. Pregnant women or women planning to become pregnant during the course of the study and nursing women 21. Patients who are knowingly hypersensitive to any of the components of the IMP or excipients 22. Patient is legally incapacitated, or persons held in an institution by legal or official order 23. Persons with any kind of dependency on the investigator or employed by the sponsor or investigator

### Trial design and safety measures

The NMS-Nab Study is a mono-centric phase II, randomized, placebo-controlled, double-blind, parallel-group, enriched enrollment withdrawal study in patients with NMS in PD (Table 2). The study includes a screening period, followed by an open-label nabilone dose optimization period (phase 1) and a placebo-controlled, double-blind, parallel-group randomized withdrawal phase (phase 2). The trial includes a screening visit (SCR), one visit after dose titration (V − 1), a baseline visit (V 0) at the beginning of phase 2 for randomization, a termination visit (V 1) at the end of phase 2, and a safety follow-up visit (V-S) 2 weeks after discontinuation of the study drug or placebo. Eligible subjects, who have signed the informed consent form, will receive open-label nabilone starting with a dosage of 0.25 milligrams (mg) in the evening after the screening visit. During dose titration period, nabilone will be titrated in 0.25-mg increments one to four times daily until a maximum dosage of 1 mg twice daily. Regular phone calls with the study center will be performed every other day during titration phase. Dose adjustments during titration phase will be performed until the patient meets the criterion of a responder (Table 3). The Clinical Global Impression of Improvement Scale (CGI-I) was chosen as a responder criterion as it represents the overall status of the patient’s improvement/deterioration. It will be assessed with regard to non-motor features of the patients.

**Table 2:** Summary of the trial registry

Data category	Information
Primary registry and trial identifying number	EudraCT: 2017-000192-86
Date of registration in primary registry	13 January 2017
Secondary identifying numbers and registry	ClinicalTrials.gov: NCT03769896 (7 December 2018, retrospectively registered) Fox Trial Finder (23 December 2018, retrospectively registered)
Primary sponsor	Medical University of Innsbruck Anichstraße 35 6020 Innsbruck Austria E-Mail: mui-sponsor@i-med.ac.at
Contact for public and scientific queries (PI and author of the study protocol)	KS, MD (Klaus.seppi@tirol-kliniken.at) Medical University of Innsbruck MP, MD (Marina.peball@i-med.ac.at) Medical University of Innsbruck
Country of recruitment	Austria
Health condition studied	Non-motor symptoms in Parkinson’s disease
Interventions	Active comparator: nabilone 0.25 mg: capsules, 0.25 mg up to 2 mg of nabilone taken orally on a daily basis Placebo comparator: corn starch, capsules, taken orally on a daily basis
Study type	Interventional Allocation: randomized Intervention model: parallel assignment Masking: double blind (subject, caregiver, investigator, outcomes assessor) Primary purpose: treatment Phase II
Date of first enrollment	December 2017
Target sample size	19 subjects per group
Recruitment status	Recruiting
Primary outcome	Change from baseline to week 4/termination visit in the MDS-UPDRS Part I (non-motor Experiences of daily living; nmEDL)
Key secondary outcomes	Change from baseline to week 4/termination visit in other assessments of motor and non-motor symptoms CGI-I scale at the termination visit
Safety and tolerability outcomes	Safety and tolerability will be evaluated with reference to the following: Tolerability, the number of subjects (%) who discontinue the study due to an adverse event (AE), the number of subjects (%) who discontinue the study due to other reasons, AEs, clinical and laboratory measurements, ECG results, vital signs, compliance, prior and concomitant medication use, different items of the MDS-UPDRS (hallucination item, orthostatic hypotension item, day-time sleepiness item), and the C-SSRS
Exploratory outcome	Changes in reaction time, attention span, and the ability to concentrate from screening to week 4/termination visit as measured by the eye-tracking examination
Ethics revision chronology	26 June 2017: Original, Protocol Version 1.2 26 January, 2018: Amendment 1 Primary reason for the amendment: Eye-tracking was added as an exploratory endpoint. A change in the list of prohibited medication was made Protocol Version 1.3 13 July 2018: amendment 2: primary reason for the amendment: the protocol was adapted to reflect changes in EU data protection regulations. Protocol Version 1.4

**Table 3:** Definition of response criteria and further steps in the trial

	Response criterion	Further steps in the trial
Criterion 1 (responder)	Patient has much (CGI-I Rating Scale: 2) or very much (CGI-I Rating Scale: 1) improved NMS on the 7-point Clinical Global Impression of Improvement Scale	Enter into the 4-week treatment period at the last prescribed dose
Criterion 2	Patient experiences intolerable side effects believed to be related to the study medication	Responder at the previous lower dose: proceed to the 4-week treatment period at the previous lower dose No responder at the previous lower dose: discontinuation Patients meeting criterion 2 at the initial dose of 0.25 mg once daily: discontinuation
Criterion 3	Patient reaches the maximum permitted dosage of 1 mg twice daily	Responder: enter the study at 1 mg twice daily No responder: discontinuation

*CGI-I* Clinical Global Impression of Improvement Scale

The open-label titration phase ends with an on-site visit (V − 1). Afterwards, patients should be on a stable nabilone dosage for at least 1 week before randomization. After at least 1 week of stable nabilone dosage, responders are randomized in a 1:1 ratio at the baseline visit (V 0) to receive either nabilone at the dosage reached during the titration phase or placebo for 4 weeks (phase 2). During the first week of the withdrawal phase, regular phone calls will be held every other day. During the open-label treatment period, dose adjustments may be performed if the CGI-I deteriorates. In this case, the patient will re-enter the titration phase of the trial.

**Fig. 1:**
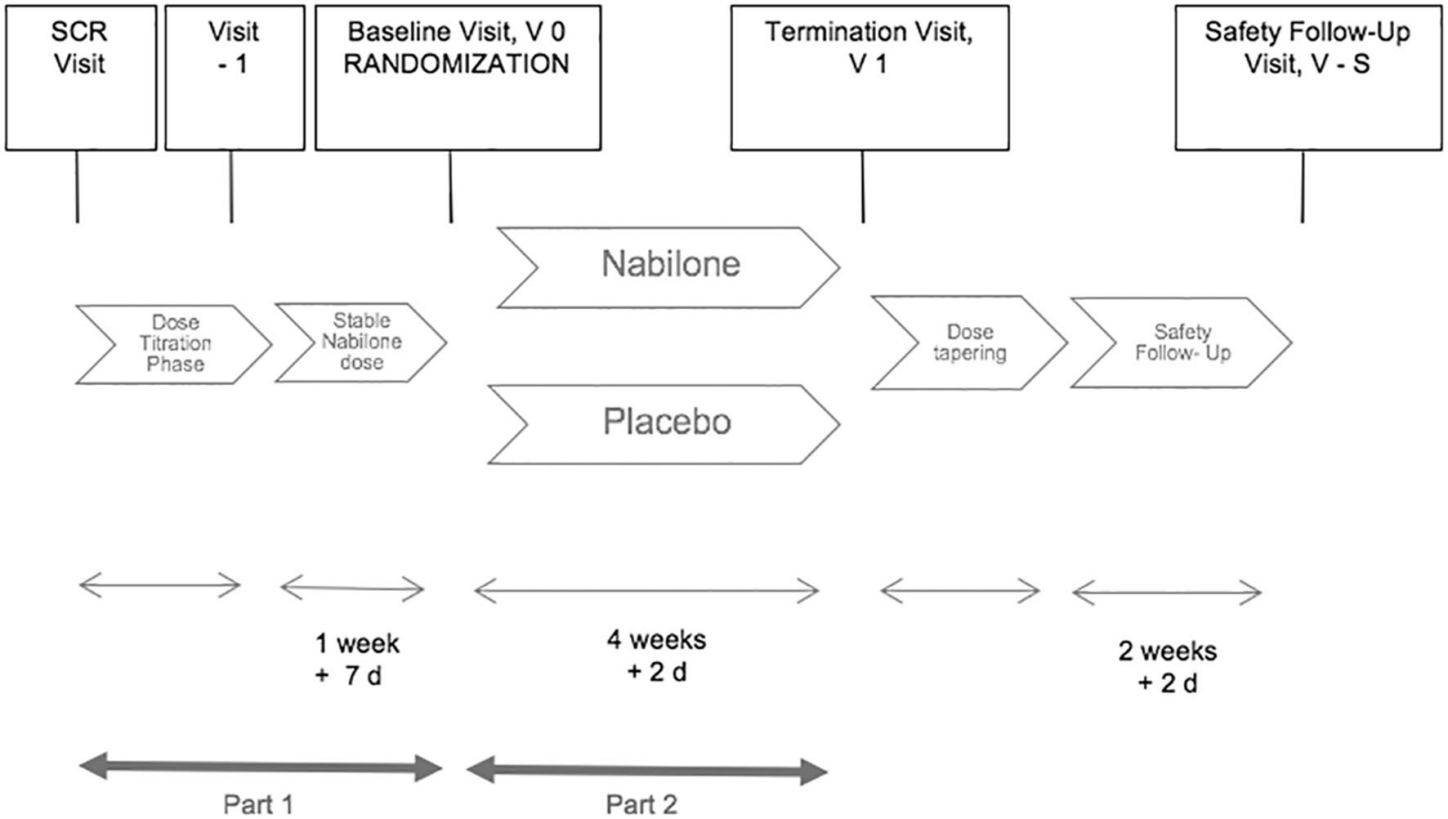
is a schematic diagram of the study procedures including all study visits and timelines

After having finished the open-label period, a termination visit will be held and nabilone will be tapered in patients in 0.25-mg twice-daily decrements. Phone calls will be held every other day during dose-tapering phase. A phone call for safety purposes will be held after 5 days and a safety follow-up visit will be scheduled after 2 weeks of discontinuation from study drug (Fig. 1).

Hypotension, fatigue, and psychosis have been described as possible side effects of nabilone therapy. Besides, in addition to suicidal tendencies, these will also be examined as safety parameters in this study in all telephone calls and visits. Moreover, laboratory measures and an electrocardiogram will be performed at screening and termination visit to monitor possible tachycardia and laboratory abnormalities.

### Randomization and unblinding

Randomization will take place in responders after up-titration of nabilone. Patients will be randomly assigned to the treatment with either placebo or nabilone at baseline in a 1:1 ratio. Randomization will be performed with a computer-generated randomization schedule provided by the Department of Medical Statistics of the Medical University of Innsbruck (MUI) in a blinded fashion for the study team and patients. The necessary medication kits are labelled with numbers according to the randomization list by the pharmaceutical company that provides the placebo and nabilone to ensure concealment. Patients will receive these kits in chronological order from blinded study team members at the baseline visit. No patient is allowed to be unblinded before the end of the trial, except for “emergencies”. Premature treatment unblinding will be performed via the emergency envelopes by a member of the study team after consultation with the principal investigator in case of an “emergency”. An “emergency” can be any event that is serious and related to the treatment in the investigator’s discretion or an event for which knowledge of the treatment group is crucial (i.e. pregnancy).

### Administrative structure, data coordinating center, study center and recruitment

The NMS-Nab Study is performed at one clinical site, the Medical University of Innsbruck (MUI, Austria, urban and rural setting) which will be the sponsor of this trial. Trained members of the study team assess the outcome measurements using validated questionnaires and clinical routine parameters. The study team undertakes the administrative and regulatory function for this trial and has access to the final trial dataset. For all work involving data collection or management of subjects, the study center will adhere to the law as laid down in the European Regulation (EU) 2016/679 as well as to the national data protection law. The study team is supported by the Clinical Trial Center of the MUI which will perform monitoring and survey the randomization process. The safety data management is performed by Hans-Günther Knaus of the Department for Medical Genetics, Molecular and Clinical Pharmacology of the MUI. Data management and statistics is conducted by qualified members of the study team of the neurological study center Innsbruck. Recruitment will last to the point when 38 subjects have finished the double-blind phase of the trial.

Patients are seen in the outpatient department on-site or at the neurologic wards. For interested patients, a member of the qualified research team will explain the study purpose, goals, and requirements in an understandable manner and an institutional review board/independent ethics committee (IRB/IEC)-approved informed consent form will be handed to the patients considering participation. These patients will be followed up by a member of the study team.

### Statistical rationale and outcomes

The primary and secondary efficacy criteria of this trial refer to the randomized, double-blind, placebo-controlled enriched enrollment randomized withdrawal phase of the study.

The primary efficacy criterion will be measured as the change of the MDS-UPDRS Part I between baseline (i.e. randomization) and week 4.

Since an interpolation of data will not be performed in case of a drop-out, the primary analysis is a per-protocol analysis. No interim analysis is planned. Secondary efficacy criteria will be measured as the change in the other clinical scales and questionnaires between baseline and week 4, except for the CGI-I measures, which will be singularly evaluated at week 4. The clinical scales include the other parts and single items of the MDS-UPDRS, the Non-Motor Symptoms Scale (NMSS), the Hospital anxiety and depression scale (HADS), the Parkinson’s Disease Questionnaire-39 (PDQ-39), the Montreal Cognitive Assessment (MoCA), the Epworth Sleepiness Scale (ESS), the Fatigue Severity Scale (FSS), the visual analog scale (VAS) of pain, the King’s Parkinson’s Disease Pain Scale (KPPS), and the QUIP-RS.

For the study’s primary efficacy and secondary efficacy objectives, mean changes from randomization to the 4-week follow-up in the nabilone and placebo groups will be analyzed separately within the two groups by Wilcoxon matched-pairs test and then compared between the two groups by Mann–Whitney *U* test. For all analyses, statistical significance will be set at the two-sided 5% level. Additionally, a sensitivity analysis will be performed for the primary efficacy or a key secondary efficacy variable, in case of differences in baseline characteristics at randomization (using Mann–Whitney *U* tests). Moreover, sensitivity to treatment will be assessed using effect sizes of the different outcome variables when using nabilone to treat non-motor symptoms in Parkinson’s disease. For CGI analyses, distributions of dichotomized ratings (amelioration, aggravation) in the nabilone and placebo groups at 4-week termination visit will be compared by Fisher exact test. The safety objectives of this study are to evaluate the safety and tolerability of nabilone in patients with PD between baseline and week 4 with reference to the number of subjects (%) who discontinue the study, the number of subjects (%) who discontinue the study due to an adverse event (AE), adverse events, serious adverse events (SAEs), clinical and laboratory assessments, assessments of vital signs including performance of active orthostatism, electrocardiogram (ECG) evaluation, patient’s compliance, patient’s prior and concomitant medication use, the hallucination item (1.2), the item for orthostatic hypotension (1.12), and the day-time sleepiness item (1.8) of the MDS-UPDRS, as well as the Columbia Suicide Severity Rating scale (C-SSRS). Distributions of AEs, SAEs, and suspected unexpected serious adverse reactions (SUSARs) in the nabilone and placebo groups at week 4 will be compared by Fisher exact test. Additionally, a descriptive analysis reporting all safety parameters mentioned above will be performed and displayed separately for the nabilone and placebo group.

To exclude that nabilone causes any negative effects on reaction time, attention span, and concentration, several tasks on an eye-tracker will be performed as an exploratory endpoint. Eye-tracking provides a fast and non-invasive method for various examinations. In this study, we will measure the reaction time using pro-saccade and anti-saccade tasks. Moreover, we will assess attention spans and the ability to concentrate using a customized pro- and anti-saccade task as well as a test involving task-switching. Mean changes from the screening visit to week 4 in error rates and reaction times in the nabilone and placebo group will be analyzed separately for the two groups by Wilcoxon matched-pairs test and then compared by Mann–Whitney *U* test.

### Sample size and power calculation

This is a phase II randomized clinical trial that uses a complete enriched enrollment randomized withdrawal design to evaluate the effects of continuous nabilone therapy versus withdrawal to placebo in patients with PD suffering from NMS. This design has the advantage that the patient population enrolled is enriched by including only responders. Moreover, total exposure to placebo in this withdrawal design may be shorter than in a study with only a randomized treatment phase. The power calculation refers to the primary endpoint of the study, i.e. change of MDS-UPDRS Part I score from randomization to week 4 during the placebo-controlled, double-blind, parallel-group randomized withdrawal phase (i.e. phase 2 of the study). A total of 38 patients (19 in each group) will have 80% power to detect a probability of 0.231 that an observation in the treatment group is less than an observation in the placebo group using a Wilcoxon (Mann–Whitney) rank-sum test with a 0.050 two-sided significance level (Table 4). With this, a statistically significant difference in the change from randomization to week 4 in MDS-UPDRS Part I score between nabilone and placebo will be detected if the true difference is 2.5 points. In the absence of previous data, we empirically chose, as clinically meaningful, a 2.5-unit change from randomization to the week 4 visit (in phase 2). This sample size calculation assumes the standard deviation of the change to be 2.4 points from randomization to week 4 (Poewe et al. 2015). Assuming a drop-out rate of 25%, we plan to include around 48 patients with Parkinson’s disease in this trial. Importantly, although a sample size calculation is provided, this is an exploratory study evaluating different NMS domains. Therefore, corrections for multiple comparisons are not planned.

**Table 4:** Estimates of sample size using different standard deviations

	1	2	3
Difference in means, *μ*1 − *μ*2	2.500	2.500	2.500
Common standard deviation, *σ*	2.400	2.600	3.000
Effect size, *δ* = (*μ*1 − *μ*2)/*σ*	1.042	0.960	0.833
Test significance level, *α*	0.050	0.050	0.050
One- or two-sided test?	2	2	2
Power (%)	80	80	80
Number per group	19	21	27
Total number of participants	38	42	54
Including 25% drop-outs	47.5	52.5	67.5

### Study setup/workflow

Permission for the conduct of the trial was received from the ethics committee of the MUI on 26 June 2017 (reference number: 1008/2017) and the Austrian regulatory authorities approved the study on the 15 September 2017. Two amendments to include an eye-tracking analysis as an exploratory endpoint in the study and to conform to the EU Data Protection Law 2018 have been approved by the ethics committee of the MUI and the regulatory authorities (Table 2). Patient recruitment was started in October 2017 and is still ongoing. The first patient was included in December 2017. The study was registered on ClinicalTrials.gov and Fox trial finder.

The results of this study will be published by study team members according to the principles of publication policy. There are no arrangements on publication issues with subsiding parties.

## Discussion

Evidence from preclinical and clinical trials suggests a rationale for the use of cannabinoids in NMS due to the influence of the ECS on processing of nociception and mood, as well as on sleep. Moreover, the overactivity of the ECS in PD patients and shared pathways of the cannabinoid and dopaminergic systems in the basal ganglia as presented in these studies justify its use in PD patients. Treatment with cannabinoids is considered to be safe and seems to be well tolerated in clinical trials and routine use in other indications. We believe that the use of cannabinoids can be an additional treatment option for symptoms not concerning motor control of PD. Data from randomized controlled trials of cannabinoids in PD are limited and mostly focus on motor abnormalities. Therefore, we have decided to perform this phase II randomized clinical trial that uses an enriched enrollment randomized withdrawal design to evaluate the effects of continuous nabilone therapy versus withdrawal to placebo in patients with PD suffering from NMS following an open-label treatment phase. The natural evolution of NMS is not well established over short durations and data on changes in MDS-UPDRS ratings are limited. The withdrawal design enrolling only responders provides an enrichment strategy for efficacy testing. Thus, the study evaluation will be based on the amelioration and/or recurrence of non-motor symptoms of PD in the randomized withdrawal phase of the study to show efficacy of the treatment (Administration  UDoHaHSFaD 2012; Biaggioni et al. 2015; Thibault et al. 2017). This is based on the assumption that if the treatment is beneficial, the withdrawal group will return to baseline values, and/or show higher drop-out rates, more adverse events, and/or a deterioration of symptom scores and CGI-I ratings compared to the treatment group. The difficulty of recruiting patients for placebo-controlled trials, high drop-out rates, and the high placebo effect experienced in clinical studies with PD patients are additional rationales for this study design (Administration  UDoHaHSFaD 2012; Biaggioni et al. 2015; Espay et al. 2015; Frisaldi et al. 2014; Thibault et al. 2017). The sample size calculation of this study was based on the assumption of a standard deviation of 2.4 points of the change from randomization to week 4. In a standard trial design, the standard deviation should be suggested to be higher due to differences in response of the patients and, therefore, a higher mean variation. With increasing standard deviation, the sample sizes will increase likewise. Assuming the standard deviation of the change of the values of MDS-UPDRS Part I between baseline and termination visit to be 2.6 points and a power of 80% with a two-sided significance level of 5% in a Wilcoxon rank-sum test, the sample size rises to 21 patients per treatment group. For a standard deviation of three points leaving all other parameters unchanged, the sample size would be 27 patients for each group. With drop-out rates of 25%, 53 patients or 68 patients would be needed in total in the randomized withdrawal phase of the trial, respectively (Table 4).

Furthermore, this trial design protects patients against long-term exposure to an ineffective treatment through early discontinuation of trial participation in case of a deterioration of the severity of the condition (e.g. CGI-I measures deteriorate) (Administration UDoHaHSFaD 2012). Moreover, individualized dosing is conceivable by the means of this trial design to reflect care in the clinical routine (Biaggioni et al. 2015). Due to different individual doses and placebo used in the trial, it can also be possible to establish a dose–response relationship (Administration UDoHaHSFaD 2012). This proof-of-concept study should be the basis for further evaluations of long-term efficacy and safety of the use of cannabinoids in PD patients in multiple clinical sites. To determine improvement or deterioration with nabilone compared to placebo in anxiety, sleep disturbances, orthostatic hypotension, and other NMS, post hoc analyses might follow the primary and secondary analyses that are defined in the actual study protocol. Although common, there is a paucity of well-performed large-scale RCTs for the treatment of the different NMS in PD (Schrag et al. 2015; Seppi et al. 2011, 2019). Unlike most motor features of PD, NMS often have limited treatment options or response (Kalia and Lang 2015). The reduction of harm through an ineffective treatment, the reduction of sample size caused by our trial design, and the possible evaluation of the concrete influence of the placebo effect on efficacy outcomes justify this design for a single-centered placebo-controlled investigator-initiated trial of nabilone. Initial data are expected in the third quarter of 2019.

## Data Availability

The dataset that is generated during the study is not publicly available because the study is currently recruiting.

## Abbreviations

AE: Adverse event
AIDS: Acquired immune deficiency syndrome
C-SSRS: Columbia Suicide Severity Rating Scale
CB: Cannabinoid
CGI-I: Clinical Global Impression of Improvement Scale
ECG: Electrocardiogram
ECS: Endocannabinoid system
ESS: Epworth Sleepiness Scale
EU: European Union
EudraCT: European Clinical Trials Database
FDA: United States Food and Drug Administration (FDA)
FSS: Fatigue Severity Scale
GABA: Gamma-aminobutyric acid
GPe: External globus pallidus
GPi: Internal globus pallidus
HADS: Hospital anxiety and depression scale
HIV: Human immunodeficiency virus
IRB/IEC: Institutional review board/independent ethics committee
KPPS: King’s Parkinson’s Disease Pain Scale
LID: Levodopa-induced dyskinesia
MDS-UPDRS: Movement Disorders Society-Unified Parkinson’s Disease-Rating Scale
mg: Milligrams
MoCA: Montreal Cognitive Assessment
MUI: Medical University of Innsbruck
NMS: Non-motor symptoms
NMSS: Non-Motor Symptoms Scale
PD: Parkinson’s diseases
PDQ-39: Parkinson’s Disease Questionnaire-39
QoL: Quality of life
QUIP-RS: Questionnaire for Impulsive-Compulsive Disorders in Parkinson’s Disease-Rating Scale
RCT: Randomized controlled trial
REM: Rapid eye movement
SAE: Serious adverse event
SCR: Screening visit
SUSARs: Suspected unexpected serious adverse reactions
THC: Tetrahydrocannabinol
V: Visit
V-S: Safety follow-up visit
VAS of pain: Visual analog scale of pain
